# The Retention Rate and Safety of JAK Inhibitors in Rheumatoid Arthritis: Real Word Data from a Monocentric Cohort

**DOI:** 10.3390/jcm13123494

**Published:** 2024-06-14

**Authors:** Denise Donzella, Elisa Bellis, Gloria Crepaldi, Valeria Data, Mariele Gatto, Claudia Lomater, Gaetano Liperoti, Elena Marucco, Marta Saracco, Annamaria Iagnocco

**Affiliations:** Academic Rheumatology Centre, Ospedale Mauriziano—Clinical and Biological Sciences Department, University of Turin, 10024 Turin, Italy; denise.donzella@gmail.com (D.D.); elisa.bellis@unito.it (E.B.); gcrepaldi@mauriziano.it (G.C.); vdata@mauriziano.it (V.D.); mariele.gatto@unito.it (M.G.); clomater@mauriziano.it (C.L.); gaetano.liperoti@edu.unito.it (G.L.); emarucco@mauriziano.it (E.M.); msaracco@mauriziano.it (M.S.)

**Keywords:** rheumatoid arthritis, JAK inhibitors, retention rate, safety, real-world data

## Abstract

**Background/Objectives**: To date, the literature concerning real-world data on the retention rate and safety of Janus kinase inhibitors (JAKis) is limited. To retrospectively evaluate the overall drug retention rate (DRR) of different JAKis in a monocentric cohort of patients with rheumatoid arthritis (RA). **Methods**: Patients diagnosed with RA and treated with JAKis who were evaluated at our outpatient clinic from March 2017 to December 2023 were included in the study. Demographic, clinical characteristics, and comorbidities were recorded. The DRR was evaluated as the time to drug discontinuation, and baseline predictors of drug discontinuation were investigated through Cox regression after adjusting for baseline confounders. **Results**: The global DRR for JAKis was 51.3%. The DRR was 37.5% for tofacitinib, 46.6% for baricitinib, 69.4% for upadacitinib, and 53.5% for filgotinib. Considering all JAKis, the only significant predictor of drug discontinuation was the use of JAKis as a first-line treatment (HR 95% CI [0.25 (0.13–0.46)]. When considering each JAKi individually, a longer disease duration predicted TOF discontinuation (HR95% CI [1.05 (1.01–1.09)], while seropositivity protected against TOF being withdrawn (HR95% CI [0.41 (0.17–0.97)]. No independent predictors emerged for other JAKis. **Conclusions**: the use of JAKis as a first-line treatment as well as disease duration and serology may impact the DRR of JAKis, which may inform tailored treatment strategies in clinical practice.

## 1. Introduction

Rheumatoid arthritis (RA) is a chronic and progressive inflammatory joint disease that can lead to severe functional impairment, a reduced quality of life, and a shortened life expectancy because of the systemic complications of both the disease itself and iatrogenic complications [1]. The introduction of biologic disease-modifying antirheumatic drugs (bDMARDs) and targeted synthetic disease-modifying antirheumatic drugs (ts-DMARDs), including Janus kinase inhibitors (JAKis), has revolutionized the management of RA. JAKis are orally administered tsDMARDs that block intracellular signaling pathways responsible for the production of pro-inflammatory molecules [2]. They have been in use in Europe and North America since 2017, when tofacitinib was the first to be approved for the treatment of RA, followed by other JAKis such as baricitinib, Upadacitinib, and filgotinib, over the years. As the scientific evidence on the long-term efficacy and safety of these drugs was gathered, the 2019 EULAR recommendations for RA management [3] included JAKis as first-line treatments alongside bDMARDs, except for patients at high thromboembolic risk, for whom caution was advised in JAKi use. However, the publication of the post-marketing ORAL Surveillance study [4], in which more major cardiovascular events (MACEs) and higher malignancy rates were observed with tofacitinib compared to TNF inhibitors (TNFi), prompted warnings from the US Food and Drug Administration (FDA) and the European Medicines Agency (EMA) [5] which were mentioned in the 2022 update to the EULAR RA Management recommendations [6]. Currently, for individuals aged 65 years or older, or those at an increased risk of major cardiovascular problems or cancer, JAKis can be used only if no suitable therapeutic alternatives are available.

However, while randomized clinical trials (RCTs) typically involve well-selected populations, real-world patients exhibit more heterogeneous characteristics and JAKis are often used for individuals who have failed multiple bDMARDs or in cases of “difficult-to-treat RA”, defined by the EULAR as persistent symptoms and/or signs of active disease after the failure of at least two b/tsDMARDs [7]. Still, the literature regarding the efficacy and safety of those drugs in a real-world setting is limited, but assessing the drug retention rate (DRR) is deemed to be a reliable measure of the safety, efficacy, and tolerability of drugs in cohorts of patients from clinical practices. In this context, we decided to analyze the overall and single DRR of JAKis and the impact of patients’ comorbidities and characteristics on them in a real-life cohort of RA patients.

## 2. Materials and Methods

### 2.1. The Study Design, Patients, and Setting

This is a an observational, retrospective, single-center study conducted at the Academic Rheumatology Centre, Mauriziano Hospital—Clinical and Biological Sciences Department, University of Turin, Italy.

Consecutive real-world patients with RA, who were followed at this center between March 2017 and December 2023, were included in the study. Patients were eligible if they met the 2010 ACR/EULAR criteria for RA [8] and were treated with a JAKi. We only included patients with complete electronic medical records, containing information such as treatment start and discontinuation dates, demographic data (age, sex), comorbidities, the date of diagnosis, the presence of rheumatoid factor (RF) and anti-cyclic citrullinated peptide antibodies (ACPA), concomitant therapies such as conventional synthetic disease-modifying antirheumatic drugs (csDMARDs) and glucocorticoids, as well as the reasons for discontinuation. Patients lacking this information were excluded from the study.

The DRR was defined as the proportion of patients who continued to use a JAKi without interruption throughout the follow-up and was assessed retrospectively. Reasons for discontinuation were categorized into three main groups: (1) primary inefficacy; (2) secondary inefficacy; (3) adverse events (including infections, skin or systemic reactions, malignancies, etc.). Adverse events were defined as “any untoward medical occurrence in a patient treated with a pharmaceutical product which does not necessarily have a causal relationship with this treatment” and were subdivided in herpes zoster infections, major adverse cardiovascular events (MACEs), malignancies, and hypersensitivity reactions [9].

The local Ethics Committee (Interagency Territorial Ethics Committee A.O.U. Città della Salute e della Scienza di Torino, Protocol No. 0005025/24) approved the study, which was conducted according to the Good Clinical Practice guidelines and the Declaration of Helsinki. Informed consent was obtained from each patient for the use of their clinical characteristics for the purposes of the study.

### 2.2. Statistical Analysis

Continuous variables were expressed as the mean ± standard deviation (SD) or the median (interquartile range, IQR) according to distribution. Comparisons between categorical variables were performed with Chi-squared tests while continuous variables were compared through Mann–Whitney U tests or Kruskal–Wallis ANOVAs with correction as appropriate. The DRR was evaluated as the time to drug discontinuation using log-rank for comparison. To assess predictors of the DRR, baseline demographic, clinical, and therapeutic variables were tested by the Cox proportional hazard model after testing for collinearity and adjusting for baseline confounders. Both uni- and multivariable analyses were performed. Stepwise regression was used to assess the variables that retained significance in multivariable analysis [9]. A threshold of 5% for alpha was set for significance.

## 3. Results

### 3.1. Clinical Characteristics

Two-hundred and thirty-six patients were included in the study. Among these, 37.3% received baricitinib, 25% upadacitinib, 23.7% tofacitinib, and 14% filgotinib. Table 1 shows the clinical characteristics of the patients for each JAKi. In total, 87.3% of patients were female, 60.2% were menopausal, 13.1% were current smokers, 78.8% tested positive for RF, 79.7% tested positive for ACPA, and 73.3% were positive for both. The mean age at diagnosis was 50.61 ± 16.85 years, the mean age and the disease duration at JAKis prescription were, respectively, 63.6 ± 12.3 and 14.8 ± 10.03 years. At baseline, erosions were found in 51.7% of cases, and interstitial lung disease was observed in 15.7%.

JAKis were administered in naive patients in 29.7% of cases, as second-line therapy in 23.7%, as third-line in 16.5%, and as fourth-line or beyond in 29.7%. The most common previously administered bDMARDs were TNF inhibitors (48.7%), followed by CTLA4-Ig (38.9%), and less frequently, IL-6 inhibitors (20.3%) and rituximab (9.7%). Only 13.6% had previously taken one or more JAKis. Furthermore, slightly less than half of the patients were taking a JAKi in monotherapy, whereas 40.7% were also receiving methotrexate (MTX), 10.6% leflunomide, 12.7% hydroxychloroquine (HCQ), and 2.9% salazosulfapiridine (SASP) (Table 2).

### 3.2. Drug Retention Rate

At the time of data collection, 51.3% of patients were receiving a JAKi, 21.1% had discontinued it due to its inefficacy, specifically, 9.7% due to its primary inefficacy (a lack of effectiveness within six months after starting) and 11.4% due to its secondary inefficacy (a loss of efficacy after six months or more), 27.6% due to adverse events (no life-threatening side effects were recorded).

In the group of patients who discontinued due to primary inefficacy, 28.6% were taking JAKis as monotherapy, while 76.2% were taking them in combination with a DMARD. In the secondary inefficacy group, 32.6% were on monotherapy, and 73% were on combination therapy. Among the group who discontinued due to adverse events, 42.19% were on monotherapy, and 59.4% were on combination therapy with a DMARD.

Ninety-three out of 236 (39.4%) patients were on JAKi monotherapy, i.e., without background cDMARDs. The duration of JAKi treatment was not statistically different between mono vs. polytherapy, neither overall nor when separating for each single JAKi. The overall median duration was 15.35 months (IQR 20.19) in monotherapy vs. 16.36 months (IQR 24.6) in polytherapy (*p* = 0.19). Withdrawal was more likely with JAKis used as monotherapy vs. polytherapy: 55 (59%) vs. 60 (42%) of patients discontinued (*p* 0.010). Particularly, polytherapy vs. monotherapy discontinuation was 40.4% vs. 72.2% (*p* = 0.005) for baricitinib 61.1% vs. 65% (*p* = 0.085) for tofacitinib, 40.2% vs. 53.8% (*p* = 0.095) for filgotinib, and 25.7% vs. 37.5% (*p* = 0.122) for upadacitinib.

**Figure 1:**
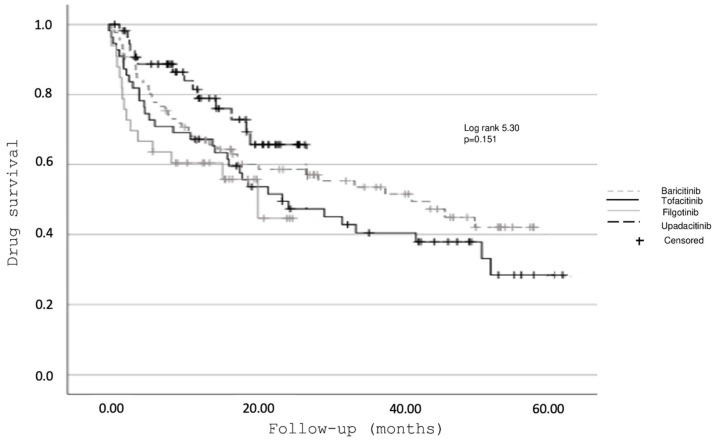
The drug retention rates of all JAKis expressed as time-to-discontinuation.

The overall median duration (IQR) of treatment with JAKis before discontinuation was 15.9 months (IQR 21.4) and varied significantly across the different molecules (Figure 1): 18.84 (37.28) months for tofacitinib, 17.42 (35.87) months for baricitinib, 8.21 (13.25) months for upadacitinib, 10.62 (14.3) months for filgotinib (*p* < 0.001).

The DRR was 37.5% for tofacitinib, 46.6% for baricitinib, 69.4% for upadacitinib, and 53.5% for filgotinib. The global DRR was 51.3%.

Table 3 shows the overall and individual JAKi DDRs by treatment lines.

Considering all JAKis together, the retention rate was significantly lower in refractory cases compared to first-line treatment [7]. In the univariable analysis, a longer disease duration, a higher number of previous treatments with bDMARDs, and previous treatment with TNFi and tocilizumab were associated with higher rates of drug discontinuation. In the multivariable Cox regression, the only significant predictor of drug retention was the use of JAKis as a first-line therapy. The prednisone daily dosage at the start of JAKi therapy, gender, age and comorbidities did not influence the DRR of JAKis in our cohort (Table 4).

**Figure 2:**
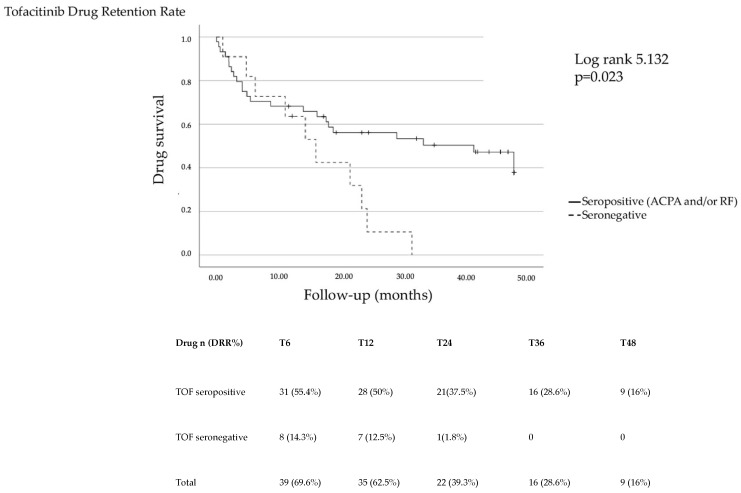
The Tofacitinib drug retention rate stratified according to seropositivity.

After adjustment for baseline confounders, independent predictors at baseline were identified for tofacitinib, for which on multivariable analysis, a longer disease duration before treatment start was found to independently predict drug discontinuation, while seropositivity (ACPA and/or RF) was found to prevent drug discontinuation (Table 5), (Figure 2).

### 3.3. Adverse Events

Table 6 shows the percentage of adverse reactions leading to discontinuation: 1.3% were herpes zoster (HZ) infections, 0.85% were MACEs, 2.1% were malignancies, and 1.69% were hypersensitivity reactions. The overall rate of HZ infection, including those that did not lead to drug withdrawal, was 6.8%. The mean age of patients who experienced these adverse events was 69 ± 8.6 years for HZ infections, 73.3 ± 4.4 years for malignancies, and 66.6 ± 6.2 years for MACEs.

## 4. Discussion

This study assessed the overall JAKi DRR and analyzed potential factors that might influence it. We found an overall cumulative DRR of JAKis to be 51.3% with no significant differences across different JAKis.

The overall median duration of treatment with a JAKi before discontinuation was 15.9 months and the reasons for discontinuation were adverse events in 27.1% of cases and ineffectiveness in 21.9%. In a recent observational cohort study [10] comparing the efficacy of TNFi, IL-6i, ABA, and JAKis in 31846 patients with RA, in terms of drug discontinuation, the results regarding the cohort of patients taking JAKis were similar to ours: the crude median drug retention was 1.19 (IQR 1.10–1.26) years and the reason for discontinuation was more often adverse events than inefficacy.

Considering all JAKis, the retention rate in our cohort was significantly lower in difficult-to-treat cases, defined according to the EULAR [7]. The only significant negative predictor of drug discontinuation in the multivariable Cox regression analysis was the use of JAKis as a first-line therapy. Across different JAKis, upadacitinib had the lowest retention in difficult-to-treat cases [7].

The univariate Cox regression analysis suggested that a longer disease duration, a higher number of previous treatments with bDMARDs, and previous treatment with TNFi could be potential factors associated with the prognostic risk of drug discontinuation. However, in the multivariable analysis, these variables did not significantly influence the DRR when considering the whole cohort. Importantly, a longer disease duration independently predicted drug discontinuation in patients receiving tofacitinib.

It is noteworthy that at the time of JAKis being started, nearly half of the patients had already failed three or more biologic drugs, while only one-third were naïve; hence, a significant portion of the population might be regarded as having “difficult-to-treat RA,” [7] with a higher risk for refractoriness. Altogether, our observations suggest that early treatment could potentially improve the DRR of JAKis.

Regarding the concomitant use of csDMARDs in our cohort, 40.7% of the patients were taking JAKis in combination with MTX, and slightly less than half of the patients were taking JAKis as monotherapy. Based on our results, the association with csDMARDs or monotherapy did not significantly affect the DRR as already reported in the existing literature [11,12], although patients on monotherapy, and particularly those on baricitinib, tended to discontinue more easily.

Another point to consider is the concomitant use of glucocorticoids. In our cohort, 71.6% of patients were taking glucocorticoids at a median (IQR) prednisone dose equivalent to 2.5 (0–5) mg which did not associate either with an improved DRR or discontinuation. With regard to adverse events, no life-threatening side effects were recorded, and a small number of MACEs, malignancies, and herpes zoster infections were observed. Although, in our study, age was not found to be related to treatment discontinuation either due to a lack of efficacy or adverse events, it is important to note that the mean age of patients who developed herpes zoster, malignancies, and MACEs in our cohort was higher than 65 years, which was set by the EMA to minimize the risk of serious side effects with JAKis [5]. In a very recently published real-world study in which the factors influencing the effectiveness and safety of the four JAKis in RA at 12 months of treatment were examined, patients aged 65 years or older showed an increased risk of JAKi treatment discontinuation due to adverse events [11].

Patients developing AE had a significantly shorter JAKi treatment duration when compared to patients undergoing withdrawal due to secondary inefficacy, while no difference was found in respect to patients withdrawing due to primary inefficacy.

Most of the patients in our cohort tested positive for either or both ACPA and RF. Compared to seronegative patients, seropositive patients for either ACPA or RF were protected against treatment discontinuation when receiving tofacitinib, suggesting that seropositivity may prompt a more timely treatment with advanced lines of therapy. To the best of our knowledge, only a small amount of data is available in the literature on patient features informing treatment strategies and conflicting observations are described on the relevance of serology in RA [11,13], which highlights the need for validation studies in wider cohorts in the pursuit of a tailored treatment.

However, the present study has some limitations. First, it is a single-center study and lacks information from other institutions. Second, for public health regulatory reasons, besides the recent EMA alert, in most cases, JAKIs were used after the failure of one or more bDMARDS. Also, unfortunately, disease activity scores were not homogeneously collected for all patients at the time of the start of a JAKi therapy. Third, because upadacitinib and filgotinib have been only recently approved for RA treatment, the number of patients treated with those drugs and included in this analysis is limited compared to the other JAKis in our study.

## 5. Conclusions

In our cohort, we found an overall cumulative DRR of 51.3% for JAKis.

Considering all JAKis, the use of JAKis as a first-line treatment protected against discontinuation. When considering each JAKi individually, a longer disease duration at baseline predicted the discontinuation of tofacitinib.

These results can provide valuable insights to enhance decision-making processes in clinical practice, although further studies with larger sample sizes and longer follow-up periods are warranted.

## Figures and Tables

**Table 1:** The clinical characteristics of the patients at baseline. Values are presented as the mean ± standard deviation or a percentage.

Clinical Features	TOF (*n* = 56)	BAR (*n* = 88)	UPA (*n* = 59)	FIL (*n* = 33)	TOT (*n* = 236)
Age at JAKi start (years)	61.7 ± 11.9	66.4 ± 12.5	60 ± 11.8	64.8 ± 11.7	63.6 ± 12
Female sex (%)	89.3	88.6	83	87.9	87.3
Disease Duration at JAKi start (years)	14.9 ± 9.9	14.8 ± 9.9	13 ± 7.9	13.5 ± 9	14.8 ± 10
RF positivity (%)	69.6	82.9	78	84.8	78.8
ACPA positivity (%)	73.2	79.5	84.7	81.8	79.7
Erosions (%)	55.4	57.9	45.8	39.4	51.7
ILD(%)	26.8	19.3	3.39	9	15.7
Hypertension(%)	42.9	46.9	35.6	39.4	41.9
Diabetes (%)	12.5	11.4	6.8	3	8.9
Dyslipidaemia (%)	21.6	20.5	16.9	9	19.9
Previous malignancy (%)	4.1	11.4	0	9	7.6

Abbreviation: RF, rheumatoid factor; ACPA, anticyclic citrullinated peptide; ILD, interstitial lung disease.

**Table 2:** The treatment features. Values are presented as percentages.

Treatment Features	TOF (*n* = 56)	BAR (*n* = 88)	UPA (*n* = 59)	FIL (*n* = 33)	TOT (*n* = 236)
Concomitant GC use (%)	76.8	70.4	61	84.8	71.6
Concomitant MTX use (%)	50	35.2	37.3	45.5	40.7
Concomitant LEF use (%)	7.1	13.6	11.8	6	10.6
Concomitant HCQ use (%)	8.9	14.8	11.8	15.1	12.7
Concomitant SASP use (%)	1.7	2.3	5	3	2.9
Naïve * (%)	19.6	31.8	37.3	23.3	29.7
Second-line treatment (%)	33.9	25	16.9	15.2	23.7
Third-line treatment (%)	8.9	18.2	20.3	18.2	16.5
Fourth-line or beyond treatment(%)	37.5	23.9	25.4	39.4	29.7
Prior TNFi use (%)	51.8	53.4	40.7	45.4	48.7
Prior aIL-6R use (%)	41	27.3	30.5	39.4	20.3
Prior CTLA4-Ig use (%)	44.6	38.6	32.2	42.2	38.9
Prior RTX use (%)	17.9	9	3.4	9	9.7
Prior JAKi use (%)	10.7	1.14	28.8	24.2	13.6
**JAKi treatment**					
Ongoing at the last observation (%)	37.5	46.6	69.4	53.5	51.3
Overall discontinuation due to inefficacy (%)	33.9	20.4	16.9	9	21.1
Discontinuation due to primary inefficacy (%)	7	9	11.9	9	9.7
Discontinuation due to secondary inefficacy (%)	28.6	10.2	5	0	11.4
Discontinuation due to adverse events (%)	26.8	34	13.6	36.4	27.6

JAKi: Janus kinase inhibitor, TOF: tofacitinib, BAR: baricitinib, UPA: upadacitinib, FIL: filgotinib, GC: glucocorticoids, MTX: methotrexate, LEF: leflunomide, SASP: salazosulfapiridine, bDMARDs: disease-modifying biological antirheumatic drugs, TNFi: tumor necrosis factor inhibitors, aIL-6R: anti-interleukin-6 receptor, CTLA4-Ig: antigen-associated cytotoxic T lymphocytes, RTX: rituximab, * naïve to both JAKi and bDMARDs.

**Table 3:** The drug discontinuation rates of all JAKis.

DDR (%)	First-Line	Second-Line	D2T	*p* Value
BAR	2.7	28.3	50	0.085
TOF	11.4	31.4	57.1	0.058
UPA	5.6	27.8	66.7	0.004
FIL	20	20	60	0.610
ALL JAKis	15.8	28.1	56.1	<0.001

**Table 4:** The Cox proportional hazard analysis for risk factors of JAK inhibitor treatment discontinuation in the overall cohort, adjusted for baseline confounders (age at diagnosis; gender; prednisone baseline dosage). Variables included in the multivariable model were disease duration, TNFi (TNF inhibitors), Tocilizumab, comorbidities, and use as a first-line therapy or D2T cases (≥2 previous bDMARDs failure).

	Univariable		Multivariable	
Covariate	HR [95% CI]	*p* Value	HR [95% CI]	*p* Value
Disease duration	1.02 (1.00–1.04)	0.035	1.02 (0.99–1.04)	0.121
ACPA	1.04 (0.55–1.96)	0.515		
RF	0.69 (0.37–1.29)	0.268		
Previous bDMARDS	1.13 (1.03–1.25)	0.012		
TNFi	1.95 (1.16–3.27)	0.013		
TCZ	2.59 (1.46–4.63)	0.001	1.83 (0.87–2.02)	0.182
Comorbidities (yes/no)	1.53 (0.88–2.61)	0.084		
JAKi as first-line treatment	0.25 (0.13–0.46)	<0.001	0.37 (0.21–0.66)	<0.001
JAKi as second-line treatment	1.58 (0.86–2.89)	0.144		
JAKi in D2T	2.16 (1.28–3.64)	0.004	1.38 (0.90–2.11)	0.140

Abbreviation: ACPA, anticyclic citrullinated peptide; bDMARDs, biologic disease-modifying antirheumatic drugs; CI, confidence interval; HR, hazard ratio; RF, rheumatoid factor; TNFi, TNF inhibitors; D2T, difficult to treat.

**Table 5:** The Cox proportional hazard analysis for risk factors of TOF treatment discontinuation adjusted for baseline confounders (age at diagnosis; gender; prednisone baseline dosage). Variables included in the multivariable model were TCZ, seropositivity, disease duration, and the use of JAKi in D2T.

	Univariable		Multivariable	
Covariate	HR [95% CI]	*p*	HR [95% CI]	*p*
Disease duration	1.05 (1.00–1.09)	0.011	1.05 (1.01–1.09)	0.006
Positive RF and/or ACPA	0.13 (0.015–1.06)	0.039	0.41 (0.17–0.97)	0.043
ACPA	0.51(0.14–1.88)	0.366		
RF	0.59 (0.17–2.04)	0.551		
Previous bDMARDS	1.12 (0.97–1.30)	0.117		
TNFi	2.43 (0.81–7.39)	0.095	1.12 (0.51–2,43)	0.784
TCZ	6.35 (1.58–25.52)	0.009	2.03 (0.97–4.23)	0.059
JAKi first line	1.26 (0.06–1.03)	0.080		
JAKi second line	0.75 (0.24–2.31)	0.166		
JAKi D2T	3.33 (1.04–10.62)	0.054	0.95 (0.40–2.42)	0.970

Abbreviation: ACPA, anticyclic citrullinated peptide; bDMARDs, biologic disease-modifying antirheumatic drugs; CI, confidence interval; HR, hazard ratio; RF, rheumatoid factor; TCZ, tocilizumab; TNFi, TNF inhibitors.

**Table 6:** The percentage of adverse reactions, leading to the discontinuation of treatment, classified as herpes zoster infections, MACEs, malignancies, and allergic reactions.

	TOF	BAR	UPA	FIL	TOT
Herpes zoster (%)	0	2.3	1.7	0	1.3
MACEs (%)	1.8	1.1	0	0	0.85
Malignancies (%)	1.8	4.5	0	0	2.1
Hypersensitivity reactions (%)	1.8	1.1	0	6	1.69

Abbreviation: MACEs, major cardiovascular events.

## Data Availability

Data are contained within the article.
